# Supplementary material for: Genomic Analyses of Wild and Cultivated Bacanora Agave (Agave angustifolia var. pacifica) Reveal Inbreeding, Few Signs of Cultivation History and Shallow Population Structure
Source: Plants (Basel). 2022 May 27;11(11):1426. doi: 10.3390/plants11111426 (PMC9183054; doi:10.3390/plants11111426)
Supplement: Supplementary file 1 [file plants-11-01426-s001.zip › plants-1704025-supplementary.pdf]

Plants

Supplementary Information for

Genomic analyses of wild and cultivated bacanora agave (*Agave angustifolia* var. *pacifica*) reveal inbreeding, few signs of cultivation history and shallow population structure

Anastasia Klimova, Karen Y. Ruiz Mondragón, Francisco Molina Freaner, Erika Aguirre-Planter and Luis E. Eguiarte

* Corresponding authors: Luis E. Eguiarte, fruns@unam.mx; Anastasia Klimova, anastasia_aleksandrovna@hotmail.com

This PDF file includes:

Tables S1 to S5

Figures S1 to S10

DNA extraction protocol

Supplementary material tables.

Table S1. Sites where individuals of *Agave angustifolia* var. *pacifica* from the state of Sonora were collected, including information on geographical region, site abbreviation, coordinates, number of individuals used for genomic analyses (*N*) and sample source (wild, cultivated, backyard plantation). Note: values of *N* do not include one individual removed during the genomic filtering step. All samples were collected in 2021.

Id	Region	Site name	Latitude	Longitude	Source	N	Elevation, m
1	Bacanora	NovW1	28°57'37.1"N	109°35'28.8"W	Wild	3	576
2	Bacanora	NovW2	28°56'51.5"N	109°30'21.1"W	Wild	3	899
3	Bacanora	NovC1	28°59'43.2"N	109°23'35.8"W	Cultivated	3	503
4	Bacanora	NovC2	28°58'42.5"N	109°24'02.4"W	Backyard	3	458
5	Bacanora	NovC3	28°56'59.9"N	109°30'53.9"W	Cultivated	3	931
6	Bacanora	NovW3	28°56'46.5"N	109°40'10.7"W	Wild	3	669
7	Mazatan	MazC1	29°12'38.4"N	110°07'20.2"W	Cultivated	3	600
8	Mazatan	MazW1	29°11'04.9"N	110°09'00.3"W	Wild	3	642
9	Mazatan	MazW2	29°10'03.5"N	110°09'17.0"W	Wild	3	749
10	Mazatan	MazC2	29°12'22.8"N	110°08'34.9"W	Backyard	3	591
11	San Carlos	SCarW1	28°18'12.1"N	111°24'02.4"W	Wild	3	34
12	San Carlos	SCarW2	27°58'37.4"N	111°07'49.2"W	Wild	3	7
13	San Carlos	SCarW3	27°56'24.3"N	111°05'51.4"W	Wild	3	66
14	Hermosillo	HermC1	29°00'49.7"N	110°57'10.6"W	Cultivated	3	276
15	Hermosillo	HermC2	29°01'20.9"N	110°57'00.2"W	Cultivated	3	249
16	Moctezuma	MocC1	29°27'34.5"N	110°16'40.8"W	Cultivated	3	422
17	Moctezuma	MocW1	29°29'32.5"N	110°11'55.8"W	Wild	3	473
18	Moctezuma	MocW2	29°46'15.4"N	109°43'24.7"W	Wild	3	807
19	Moctezuma	MocW3	29°48'12.8"N	109°38'20.7"W	Wild	3	674
20	Moctezuma	MocC2	29°41'08.3"N	109°38'05.9"W	Cultivated	3	615
21	Moctezuma	MocC3	29°27'34.5"N	110°16'40.8"W	Cultivated	3	422
22	Navojoa	NavW1	26°44'11.7"N	109°34'09.5"W	Wild	3	4
23	Navojoa	NavW2	26°46'06.0"N	109°28'47.2"W	Wild	3	8
24	Navojoa	NavW3	26°49'11.1"N	109°17'18.5"W	Wild	3	56
25	Navojoa	NavC1	27°04'29.9"N	109°20'41.4"W	Cultivated	3	80
26	Navojoa	NavC2	27°05'28.7"N	109°06'18.9"W	Cultivated	3	274
27	Navojoa	NavC3	27°05'28.7"N	109°06'18.9"W	Cultivated	3	274
28	Navojoa	NavC4	27°02'33.4"N	108°58'50.8"W	Cultivated	3	440

29	Cam (Carretera Hermosillo-Guaymas)	CamW	28°28'38.4"N	111°02'43.7"W	Wild	3	208
30	Yecora	YecW1	28°49'14.2"N	110°33'13.7"W	Wild	2	447
31	Yecora	YecW2	28°34'03.7"N	109°41'37.6"W	Wild	2	608
32	Yecora	YecW3	28°34'21.3"N	109°33'09.1"W	Wild	1	188
33	Yecora	YecW4	28°29'17.0"N	109°22'09.7"W	Wild	2	858
34	Yecora	YecW5	28°25'57.6"N	109°14'54.1"W	Wild	2	645

Table S2. Quality information for the filtered SNPs used in the present study. The data shown is for 95 individuals of *Agave angustifolia* var. *pacifica* from the state of Sonora, Mexico, and 11, 619 SNPs.

Quality statistic	Mean (SD)
Individual depth	48.6 (6.5)
Individual missingness	0.028 (0.01)
Site missingness	0.028 (0.029)

Table S3. Descriptive genomic diversity estimates and corresponding confidence intervals in parentheses as determined for each management type. N—number of samples; MLH—multilocus heterozygosity; sMLH—standardized multilocus heterozygosity; F_{IS} -Wright's inbreeding index; Fhat3—inbreeding index; IR—internal relatedness.

Management	N	MLH	sMLH	F_{IS}	Fhat3	IR
Wild	53	0.22 (0.002)	0.99 (0.013)	0.13 (0.012)	0.13 (0.012)	0.05 (0.010)
Cultivated	42	0.22 (0.003)	1.00 (0.009)	0.13 (0.007)	0.13 (0.007)	0.05 (0.006)

Table S4. Descriptive genomic diversity statistics and corresponding confidence intervals in parentheses for *Agave angustifolia* var. *pacifica* from the state of Sonora, Mexico based on population and management type. N—number of samples; MLH—multilocus heterozygosity; F_{IS} -Wright's inbreeding index; Fhat3—inbreeding index; IR—internal relatedness. Region/population column is coded according to Table S1.

Region/Population	N	Management	MLH	F_{IS}	Fhat3	IR
Novillo	9	Wild	0.22 (0.015)	0.15 (0.058)	0.15 (0.066)	0.07 (0.052)
Mazatan	6	Wild	0.23 (0.011)	0.09 (0.045)	0.11 (0.041)	0.04 (0.037)
San Carlos	9	Wild	0.22 (0.002)	0.14 (0.009)	0.13 (0.010)	0.06 (0.010)
Moctezuma	8	Wild	0.22 (0.002)	0.13 (0.009)	0.13 (0.012)	0.05 (0.009)
Navojoa	9	Wild	0.22 (0.008)	0.13 (0.319)	0.11 (0.027)	0.04 (0.029)
Camino (Cam), (Carretera Hermosillo-Guaymas)	3	Wild	0.22 (0.015)	0.15 (0.058)	0.15 (0.033)	0.08 (0.051)
Yecora	9	Wild	0.22 (0.006)	0.14 (0.025)	0.13 (0.021)	0.05 (0.023)
Hermosillo	6	Cultivated	0.22 (0.010)	0.15 (0.040)	0.15 (0.046)	0.06 (0.036)
Mazatan	6	Cultivated	0.22 (0.008)	0.12 (0.032)	0.12 (0.016)	0.04 (0.016)
Moctezuma	9	Cultivated	0.22 (0.004)	0.12 (0.016)	0.13 (0.011)	0.05 (0.010)
Navojoa	12	Cultivated	0.22 (0.003)	0.13 (0.012)	0.13 (0.014)	0.06 (0.008)
Novillo	9	Cultivated	0.22 (0.003)	0.12 (0.013)	0.12 (0.014)	0.04 (0.010)

Table S5. Number of loci and raw SNPs recovered for the datasets under different ipyrad clustering parameters.

Samples	Clustering parameter	Loci	SNPs
All sites	0.85	86,014	610,119
	0.9	94, 174	638,704
	0.95	111,390	598,158

Supplementary material figures.

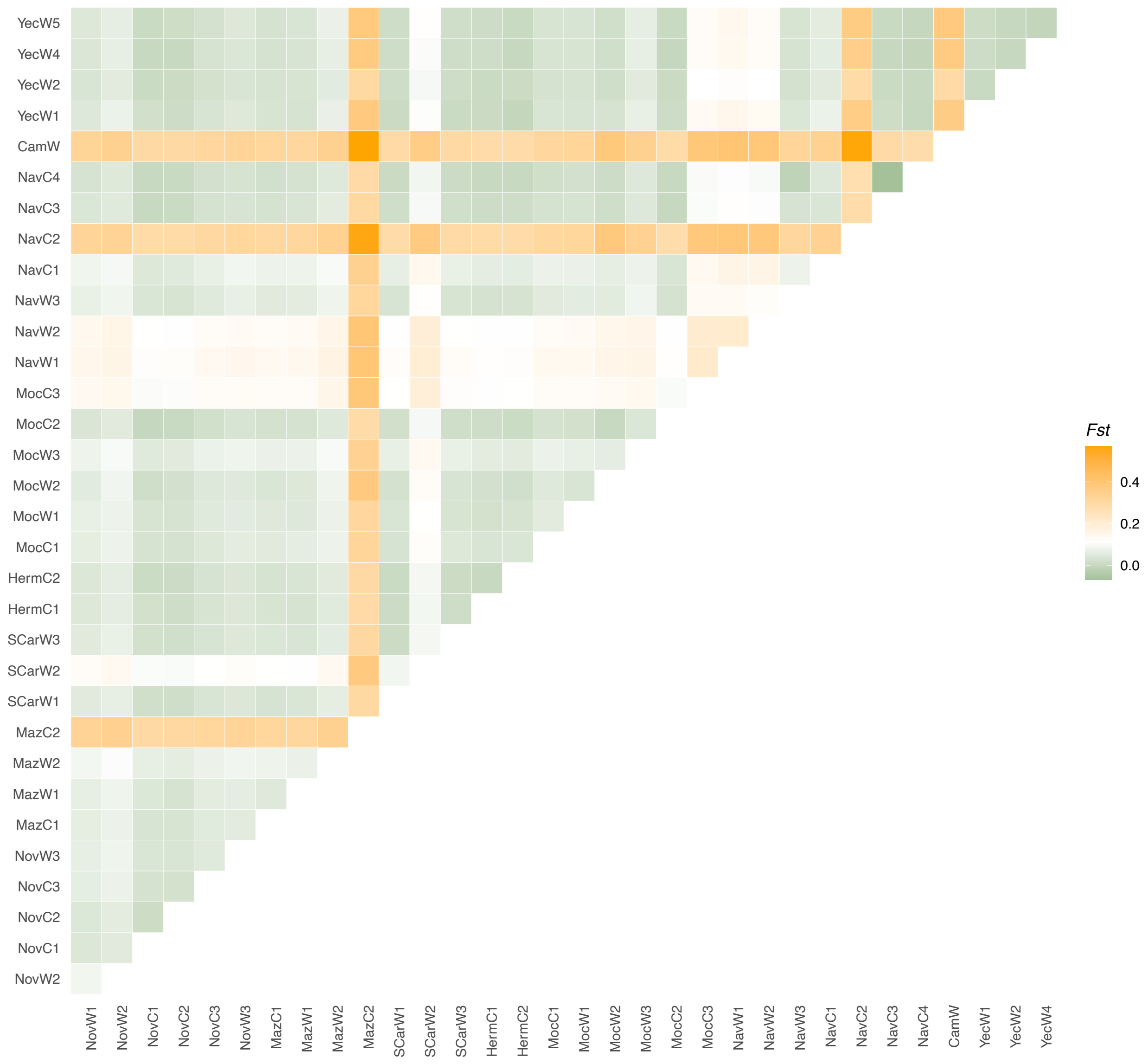

Figure S1. Pairwise F_{ST} differences among all the sampling sites of wild and cultivated *Agave angustifolia* var. *pacifica* from the state of Sonora, Mexico. Colors represent F_{ST} values from the lowest of −0.07 in green to the highest of 0.57 in orange. Sampling sites are coded according to Table S1.

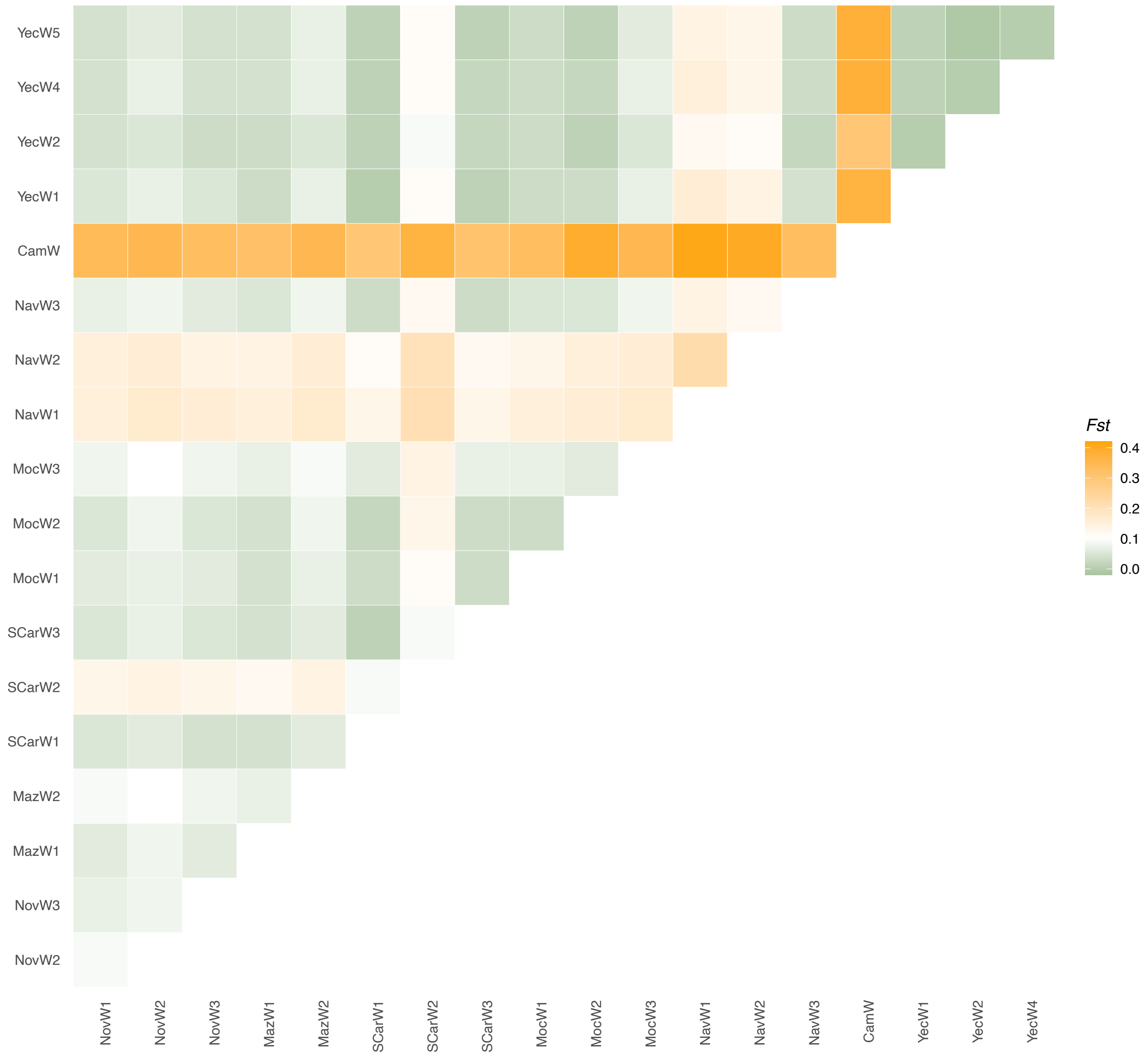

Figure S2. Pairwise F_{ST} differences among all the sampling sites of wild *Agave angustifolia* var. *pacifica* from the state of Sonora, Mexico. Colors represent F_{ST} values from the lowest of −0.01 in green to the highest of 0.41 in orange. Sampling sites are coded according to Table S1.

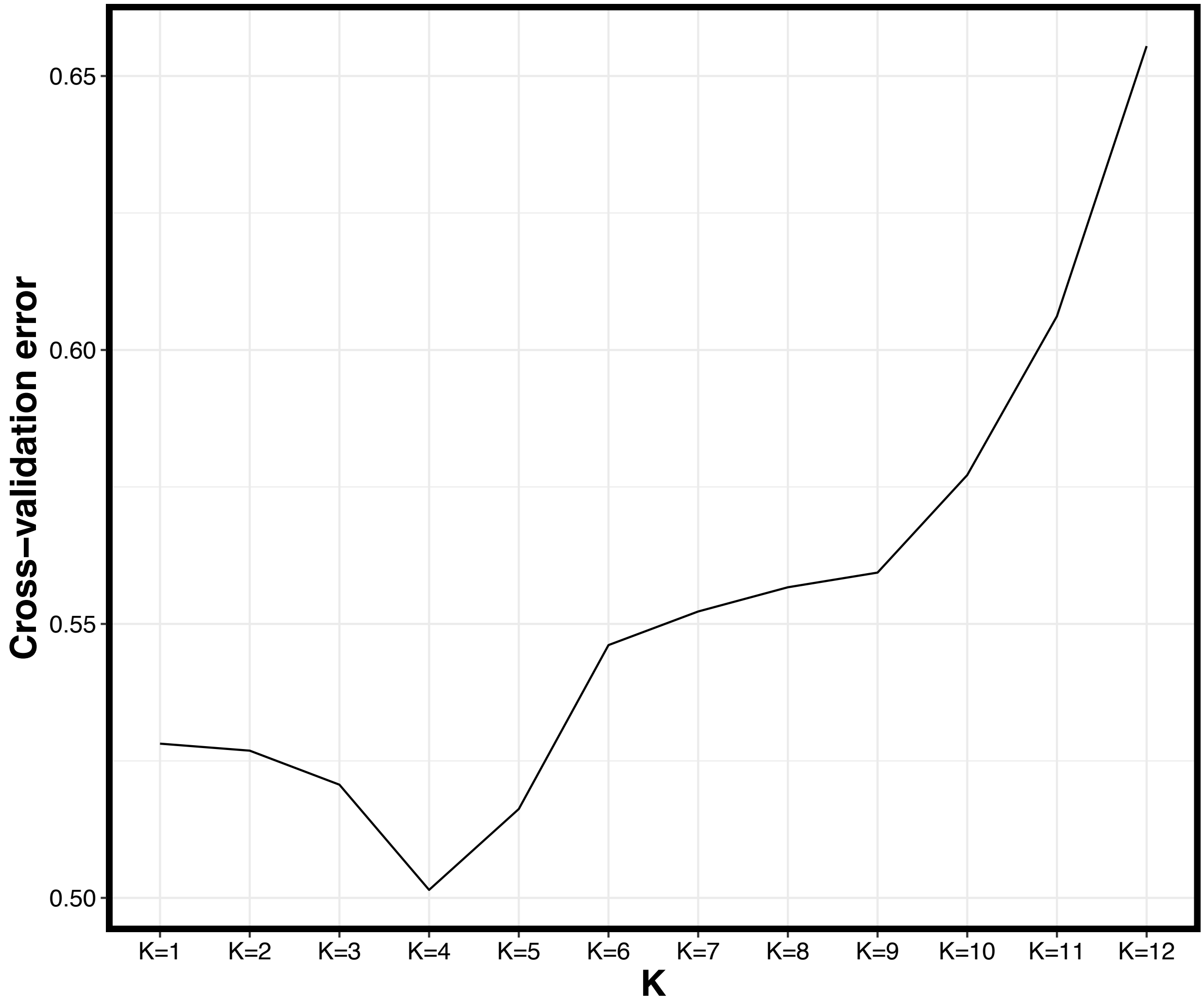

Figure S3. Plot of ADMIXTURE cross validation error from K = 1 through K = 12, as based on all samples (cultivated and wild).

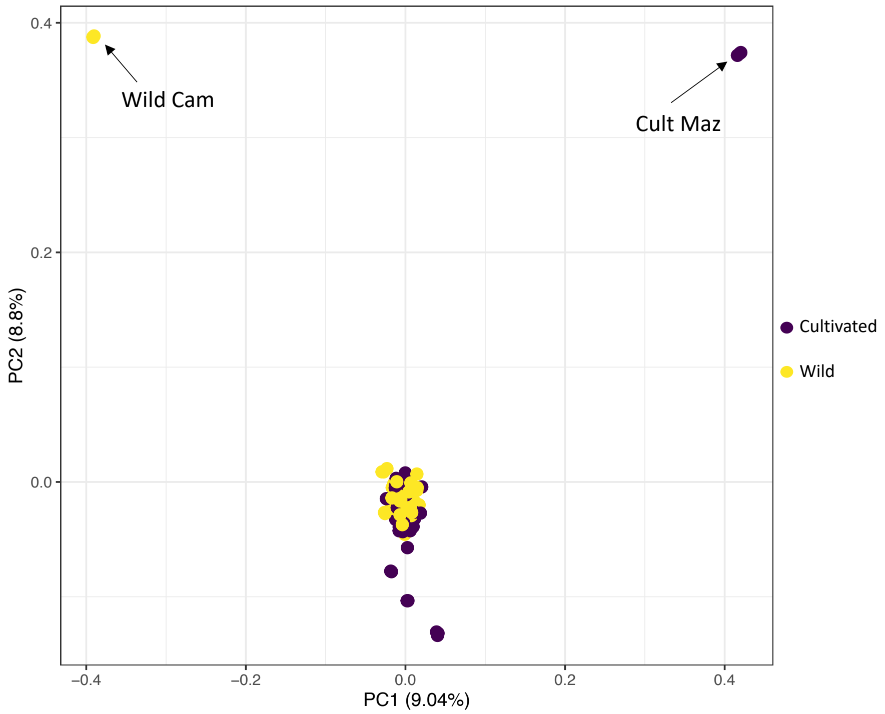

Figure S4. Relationships among *Agave angustifolia* var. *pacifica* individuals from the state of Sonora, Mexico, wild and cultivated samples as represented by principal component analysis (PCA) using 11,619 genome-wide SNPs. All 95 samples were included.

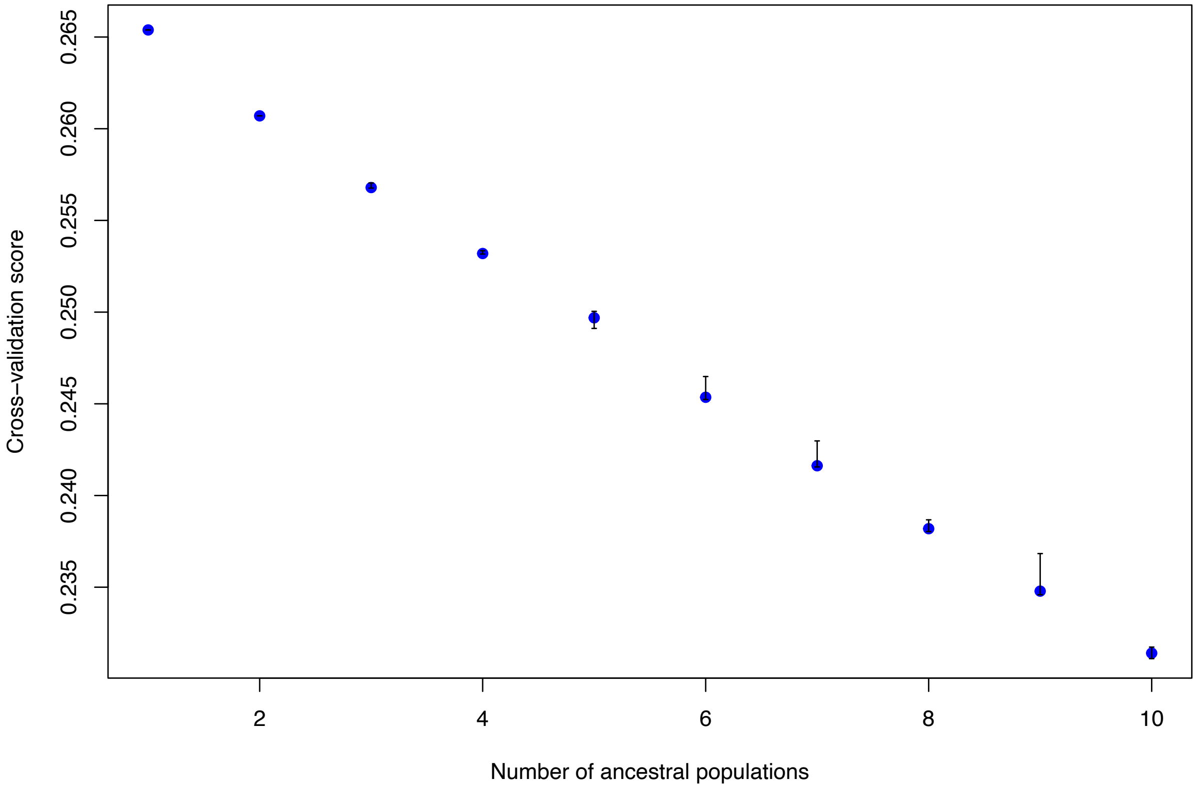

Figure S5. Plot of TESS3 cross validation error from $K = 1$ through $K = 10$, based only on wild samples. Twenty runs were performed for each value of K.

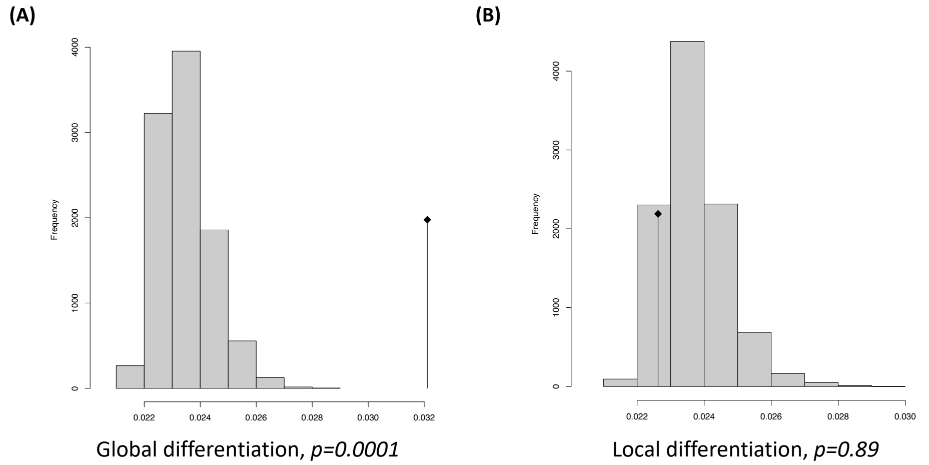

Figure S6. Results of eigen value test for sPCA analysis. The (A) global differentiation is signifi cant when compared to the (B) local differentiation. The figure was generated using the R packag e *adegenet*.

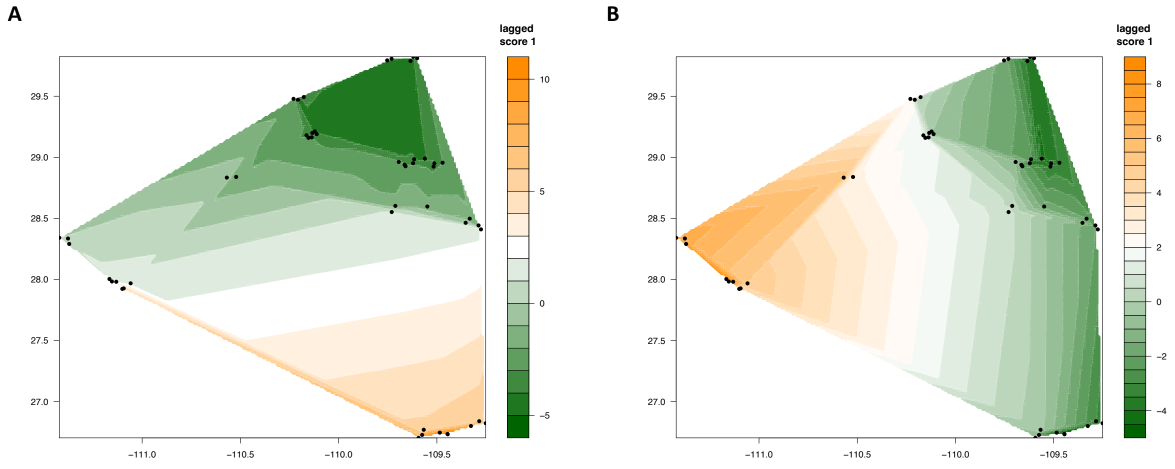

Figure S7. Results of the spatial PCA analysis; lagged scores are plotted as a cline map, panel (A) representing the first axis and (B) representing the second axis. Black dots correspond to sampling sites.

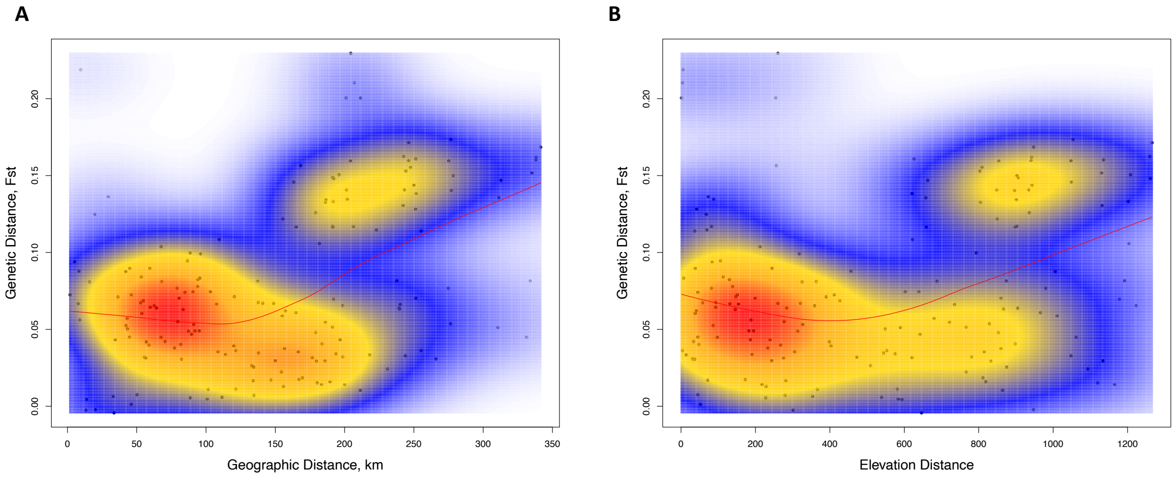

Figure S8. Results of the (A) isolation by distance and (B) isolation by elevation plots illustrating the relationships between genetic differentiation among sites of wild *Agave angustifolia* var. *pacifica* from the state of Sonora, Mexico, and (A) geographic distance (r = 0.41, p = 0.007) and (B) isolation by elevation (r = 0.19, p = 0.01), using a two-dimensional kernel density estimation as implemented in the *MASS* package in R.

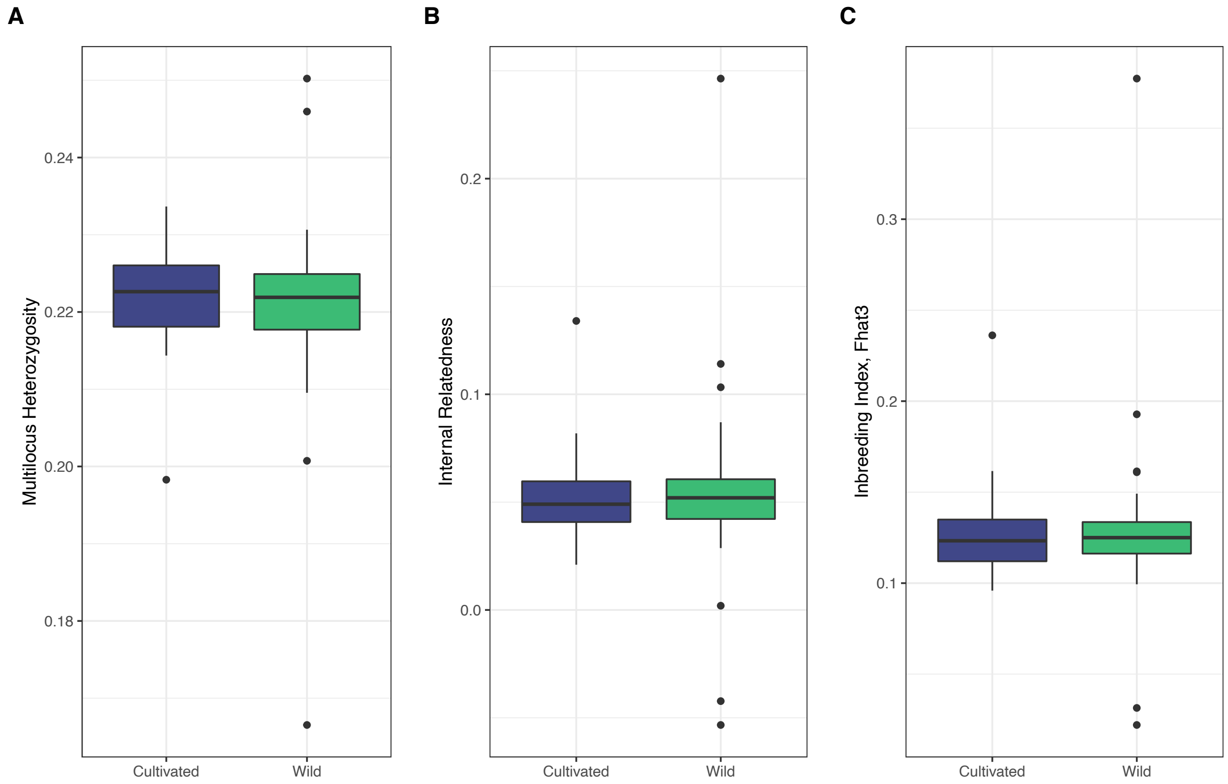

Figure S9. Boxplots of the individual based diversity estimates and inbreeding index for wild (green) and cultivated (blue) samples of *Agave angustifolia* var. *pacifica* from the state of Sonora, Mexico. No significant differences were found.

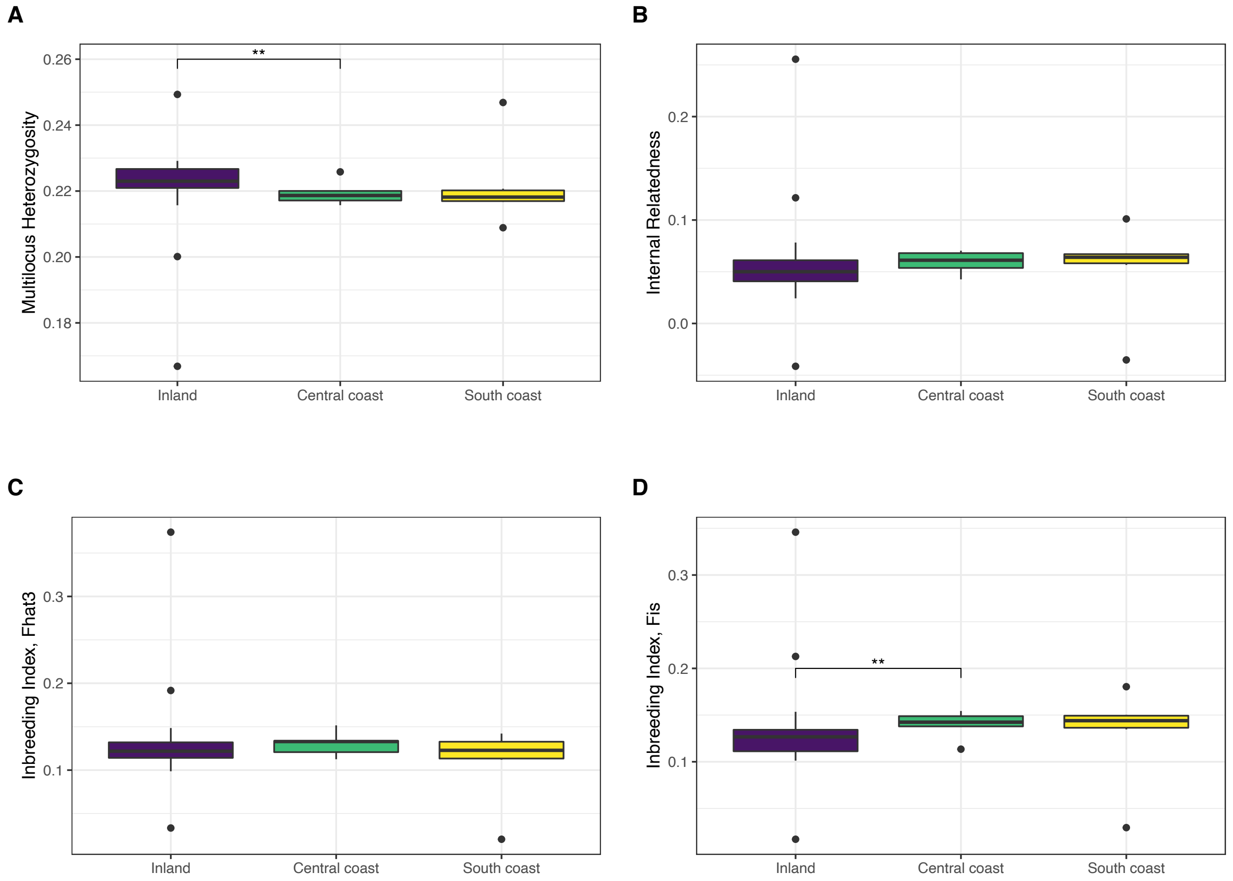

Figure S10. Boxplots of the individual based diversity estimates and inbreeding index for wild *Agave angustifolia* var. *pacifica* from the state of Sonora, Mexico populations identified with spatial analysis. Significant differences between groups (after FDR correction) are represented by horizontal black line connecting groups that were significantly different, with asterisks corresponding to the significance level (* < 0.05, ** < 0.001 and *** < 0.0001).

Detailed DNA extraction protocol.

CTAB buffer:

- CTAB buffer 2% CTAB X (hexadecyltrimethylammonium bromide)
- 100 mM TrisHCl [pH=8]
- 20 mM EDTA
- 0.2% β-mercaptoethanol (added just before use)

Steps:

1. Grind the tissue with liquid nitrogen (from 0.1g to 1g) and add 600 µL of CTAB2X. Transfer to a 1.5 ml microtube.
2. Spin at 10 000 rpm for 8 min at 4ºC.
3. Discard the aqueous phase and resuspend with 600 µL of CTAB2X buffer. Add 5 µL of RNase (10mg/ml).
4. Incubate a 65ºC for 15 min. Mix by inverting the microtube from time to time. Afterwards keep in ice for 10-15 min.
5. Add 600 µL of chloroform:octanol (24:1) solution - work in the fume hood. Gently mix by inverting the microtube.
6. Spin at 13 000 rpm for 15 min at 4ºC.
7. Carefully transfer the aqueous phase (above the white interface layer) to a clean microtube (then discard the rest).
8. Add 2.5X the volume of absolute ethanol and 1/10 the volume of cold sodium acetate. Incubate overnight at 4ºC.
9. Spin 13000 rpm for 10 min at 4ºC and discard the supernatant.
10. Add 1 ml of cold 70% EtOH and spin at 13000 rpm for 10 min at 4ºC. Repeat this step once or twice to wash the pellet.
11. Remove the supernatant and dry the pellet by living tube open at room temperature.
12. Resuspend the pellet in 100-200 sterile water 100-200ul
